# Brain-inspired modular echo state network for EEG-based emotion recognition

**DOI:** 10.3389/fnins.2024.1305284

**Published:** 2024-03-01

**Authors:** Liuyi Yang, Zhaoze Wang, Guoyu Wang, Lixin Liang, Meng Liu, Junsong Wang

**Affiliations:** ^1^College of Big Data and Internet, Shenzhen Technology University, Shenzhen, China; ^2^School of Engineering and Applied Science, University of Pennsylvania, Pennsylvania, PA, United States; ^3^Department of Auromation, Tiangong University, Tianjin, China

**Keywords:** modular echo state network, emotion recognition, EEG, memory capacity, heterogeneity

## Abstract

Previous studies have successfully applied a lightweight recurrent neural network (RNN) called Echo State Network (ESN) for EEG-based emotion recognition. These studies use intrinsic plasticity (IP) and synaptic plasticity (SP) to tune the hidden reservoir layer of ESN, yet they require extra training procedures and are often computationally complex. Recent neuroscientific research reveals that the brain is modular, consisting of internally dense and externally sparse subnetworks. Furthermore, it has been proved that this modular topology facilitates information processing efficiency in both biological and artificial neural networks (ANNs). Motivated by these findings, we propose Modular Echo State Network (M-ESN), where the hidden layer of ESN is directly initialized to a more efficient modular structure. In this paper, we first describe our novel implementation method, which enables us to find the optimal module numbers, local and global connectivity. Then, the M-ESN is benchmarked on the DEAP dataset. Lastly, we explain why network modularity improves model performance. We demonstrate that modular organization leads to a more diverse distribution of node degrees, which increases network heterogeneity and subsequently improves classification accuracy. On the emotion arousal, valence, and stress/calm classification tasks, our M-ESN outperforms regular ESN by 5.44, 5.90, and 5.42%, respectively, while this difference when comparing with adaptation rules tuned ESNs are 0.77, 5.49, and 0.95%. Notably, our results are obtained using M-ESN with a much smaller reservoir size and simpler training process.

## 1 Introduction

Emotions play an essential role in the human decision-making process (Picard, 1997). Automated recognition of emotions has broad applications in improving human-computer interactions (Atkinson and Campos, 2016), facilitating the diagnosis and treatment of affective disorders (Cai et al., 2020), and helping content providers enhance user experiences. Traditionally, emotional recognition relies on non-physiological cues such as facial expressions, speech, and behavior. However, subjects can inhibit these reactions, thus causing inaccurate classification results (Mauss and Robinson, 2009). Emotion recognition based on physiological activities such as EEG signals can circumvent this limitation.

Nonetheless, EEG is a complex signal that often requires advanced computational models to decode. Recently, a great number of machine learning and deep learning techniques have been applied to interpret EEG signals for emotion recognition (Li et al., 2022). Zheng and Lu (2015) utilized deep belief network and support vector machine to classify positive, negative, and neutral emotions. RNN frameworks, such as Long Short-Term Memory (Alhagry et al., 2017) and Spatial–Temporal RNN (Zhang et al., 2018), are also widely employed as the RNN structures are natural fits for sequential data like EEG signals. Convolutional neural networks (CNNs) (Lin et al., 2017; Chen et al., 2019) and hybrid architectures (Li et al., 2016) also obtained remarkable results. However, several challenges remain: (1) CNN-based architectures, despite their exemplary performance in detecting spatial information, their feed-forward design makes it less efficient in leveraging complex long-distance temporal patterns. (2) On the other hand, RNN-based models trade off their time and space complexity to mitigate the vanishing gradient problems. Therefore, it is challenging to implement them on wearable devices where computational resources are limited.

**Figure 1:**
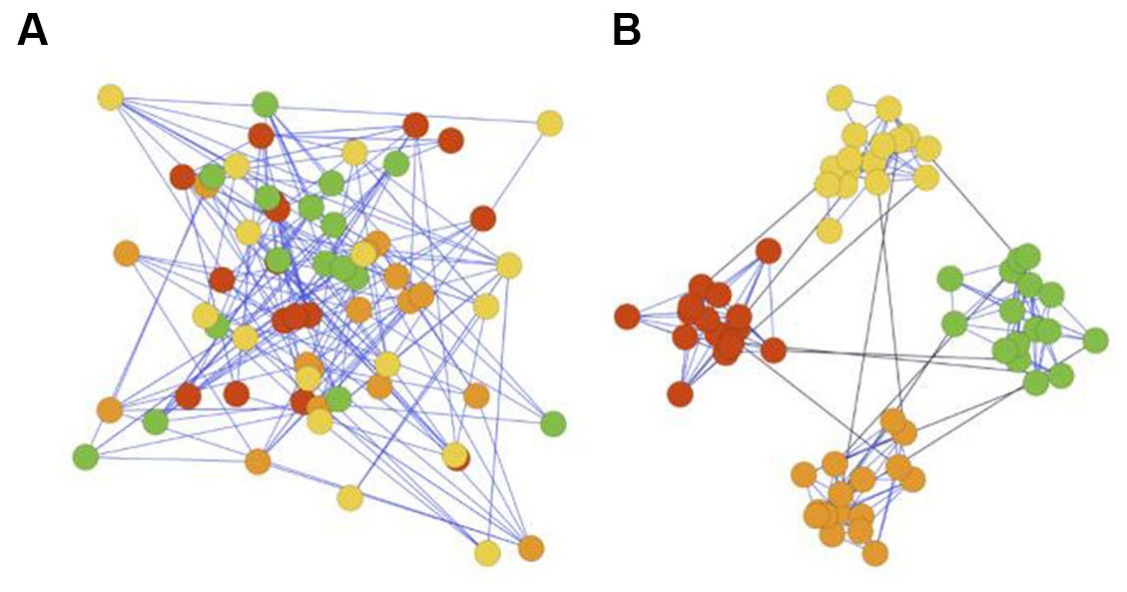
**(A)** Network without community structures. **(B)** Network with community structure.

A special category of RNN called Echo State Network (ESN) (Jaeger, 2001a,b) offers a promising solution to the above-mentioned shortcomings. ESN contains a reservoir layer that simulates brain synaptic connections using a randomly connected network (see Figure 1A). The random connections non-linearly project the input into the internal states of the reservoir, making ESN particularly powerful on many time-series prediction and classification tasks. However, the reservoir layer is generally untrained due to the limitation of backpropagation on circular connections; only the last layer (i.e., the readout layer) is trained on the input data. This configuration allows ESN to exploit long-distance dependencies while maintaining exceptional computational simplicity, yet it also makes ESN performance particularly vulnerable to random initialization. In previous studies using ESN to decode emotional information in EEG signals, neural adaptation rules such as intrinsic plasticity (IP) (Schrauwen et al., 2008) and synapses plasticity (SP) (Oja, 1982; Castellani et al., 1999) were used to alleviate this deficiency. IP rule maximizes the information entropy in the reservoir by adjusting the activation function of the reservoir, while SP rules update the connections weights to make reservoir dynamics more stable.

Existing attempts using ESN for EEG-based emotion recognition are 2-fold. The first kind adopts ESN as EEG feature selectors (Koprinkova-Hristova et al., 2015; Bozhkov et al., 2016). In this setting, an IP-tuned ESN takes EEG signals to generate a lower-dimensional representation, while a clustering algorithm was applied to the *n*-leading lower-dimensional features to classify emotions. Bozhkov et al. (2017) showed that IP-tuned ESNs outperform regular ESNs because they can yield more separable features in the hyper-plane. On the other hand, the second type adopts ESN directly as classifiers (Fourati et al., 2017), in which ESNs predict emotion labels in an end-to-end manner. Several adaptation rules such as IP, anti-Oja, and BCM rule were employed on ESN (Fourati et al., 2020b) for better network robustness. An ESN with leaky integrators and multiple reservoir layers called Leaky DeepESN-IP (Fourati et al., 2020a) was also investigated to mitigate the impacts of inter-subject variability.

Nonetheless, we argue that these ESN methods can be further improved. ESN resembles the biological brain using a random network. Since biological brains are far from being entirely random (Bullmore and Sporns, 2009; Bullmore and Sporns, 2012; Tripathy et al., 2013), this over-simplification may hamper ESN performance. Moreover, even though adaptation rules are readily available, their algorithm and training procedures are complex. In an effort to resolve the above limitations, there are several pioneering works that attempt to initialize ESN to a predefined topology. Deng and Zhang (2007) proposed scale-free highly clustered ESN, an ESN with neurons distributed in a scale-free structure. Similarly, Li et al. (2016) generated a multi-clustered structure by first placing pioneering nodes in a three-dimensional space, then adding new nodes and connecting them to their closest neighbor based on their Euclidean distance. Rodriguez et al. (2021) initialized ESN to a two-module structure for optimal information diffusion. They demonstrated that for ESN using step-activation functions, there exists an optimal level of modularity that will maximize the model performance on the memory capacity task and the recall task.

The main advantage of ESN model is its simple structure, and there is no need to train the weights inside the reservoir. However, the general ESN network mainly adopts a random structure, which is not a structure with optimal performance. In order to further improve the performance of ESN, the weights inside the reservoir have been trained in some studies, including synaptic plasticity as well as intrinsic plasticity methods. Although these methods can improve the performance of ESN, they also increase the complexity of training. Modularity is a prevalent structure in brain neural networks, which is the result of the evolution and optimization of brain neural networks; therefore, this study directly draws on the modular structure of brain networks, which promotes the performance of ESN, while the design is relatively simple.

Motivated by several recent findings indicating brain modularity improves cognitive abilities (Crossley et al., 2014; Bertolero et al., 2018), in this proposed work, we continue to explore the possibility of adopting modular structure in ESN. Differing from the previous study (Rodriguez et al., 2021), which uses a single parameter to optimize the modular structure, we propose a novel triple-parameter optimization method for modular ESN. Our method allows flexible control over the module number, intra-module connectivity, and inter-module connectivity. We present a comprehensive experimental analysis of how each of these parameters impacts network performance. The proposed Modular Echo State Network (M-ESN) demonstrates significant performance improvement on the DEAP benchmark for EEG emotion classification without any neural adaptation procedure. In summary, our major contributions are as follows:

- We propose a bio-inspired lightweight framework for EEG classification, namely M-ESN. We present a novel triple-parameter method for defining and optimizing the modular topology.
- On the DEAP benchmark, our proposed M-ESN with 600 neurons outperformed the neuron-adaptation-rule-tuned ESN with 1,500 neurons by 0.77, 5.49, and 0.95% on the arousal, valence, and stress/calm classification tasks.
- We provide explanations for the modularity enhancing ESN performance from several perspectives. We found that the increasing modularity will induce changes in network topology. We show that these topological changes increase network heterogeneity and thereby improve the information processing efficiency.

## 2 Related works

### 2.1 Modularity in biological neural network

Modular structure, also referred to as community structure, describes networks in which neurons are more interconnected within their own cluster and less connected to neurons in other clusters (see Figure 1B). Modular structure is found in the neuronal system of many species, ranging from nematode *C*. *elegans* to mammals (Kim and Kaiser, 2014), suggesting it may confer genetic advantages.

This structure is also confirmed by well-establishing neuroscientific evidence (Sporns et al., 2005; Meunier et al., 2010; Sporns and Betzel, 2016). Structural evidence shows neurons within the same anatomical region in human brains are densely connected by synapses, while long-distancing white matter tracts sparsely connect these segregated regions to enable interregional information transfer (Avena-Koenigsberger et al., 2017). Moreover, the functional modeling of the human brain, which characterizes neuronal dynamics, also appears to be modular (Zhou et al., 2006). Finally, various anatomical regions typically have diverse gene expressions (Krienen et al., 2015), indicating brain is evolved in a modular manner to support function specialization (Bassett et al., 2010; Taylor et al., 2017; Figure 1A).

### 2.2 Modularity promotes network efficiency

Emerging neuroscientific findings suggest modular network promotes network efficiency. Crossley et al. (2014) investigated the modularity in brain functional coactivation networks, in which they concluded that brain modules promote cognitive specialization. Bertolero et al. (2018) offered indirect evidence that brain modularity enhances cognitive abilities. They investigated the role of hub structure, which is ubiquitous in modular networks (Bullmore and Sporns, 2009), on cognitive ability. They indicated that individuals with more diversely connected hubs perform universally better on several cognitive tasks. Lastly, several studies have reported that modular structure promotes the reuse of recurring network patterns (Kashtan and Alon, 2005) and network adaptability (Clune et al., 2013).

To summarize, existing literature mainly attributes modular topology to (1) leveraging the brain wiring costs and the efficacy of the information propagation (Betzel et al., 2017); (2) providing functional versatility and adaptability (Bassett et al., 2011); and (3) enabling structural flexibility that allows the neural system remain unaffected by local modifications (Kim and Kaiser, 2014). Motivated by these findings, we adopt the modular structure in ESN to improve the model performance.

### 2.3 EEG-based emotion recognition using hybrid CNN and LSTM classification

Electroencephalography (EEG)-based emotion classification is an important research area in emotion recognition, with a significant challenge being the individual differences and temporal variability in EEG recordings. Shen et al. (2020) proposed a novel four-dimensional convolutional recurrent neural network method to address this issue, effectively integrating frequency, spatial, and temporal information to overcome the interference caused by individual and temporal variability, thereby significantly improving the accuracy of emotion recognition. However, this method may face issues of high model training complexity and computational costs. Zhang et al. (2020) explored the potential of emotion recognition using multiple deep learning architectures, and validated the effectiveness of the CNN-LSTM hybrid model in processing EEG signals. This model successfully leveraged the advantages of CNN in feature extraction and the ability of LSTM in handling long-term dependencies in time-series data, but it may also lead to overfitting, especially with limited data. Additionally, Zamani and Wulansari (2021) and Samavat et al. (2022) presented models based on 1D-CNN, RNN, and a multi-input hybrid model based on CNN and Bi-LSTM, focusing on the analysis of specific frequency and temporal features. They enhanced the performance of emotion recognition by capturing temporal information more comprehensively through bi-directional LSTM, despite the high complexity in model structure design and the need for a large amount of annotated data for training. As for the hybrid emotion model proposed by Patlar Akbulut (2022), the utilization of transfer learning with large-scale sensor signals further improved the accuracy of emotion classification, highlighting the advantages of multimodal data and transfer learning in overcoming subject differences and data scarcity issues. Cimtay and Ekmekcioglu (2020) focused on the utilization of pre-trained CNN models, exploring the potential of cross-subject and cross-dataset emotion recognition, thus avoiding the resource consumption of training models from scratch. However, pre-trained models may face challenges in transfer efficiency and fine-tuning precision.

## 3 Methods

### 3.1 Dataset

We used the famous DEAP benchmark (Koelstra et al., 2011) on our model to provide fair comparison results with other existing methods. The DEAP dataset is collected when 16 male and 16 female participants watching a collection of 40 music videos. While the subjects are watching video clips, 32 channels of EEG signals and eight channels of peripheral signals are collected at 512 Hz and down-sampled to 128 Hz. Only the first 32 channels, i.e., the EEG channels, are used in our study. Each video clip lasts 63 s. The first 3 s of pre-trail baseline are removed for all trails. Therefore, each trail has a size of 60 × 128 × 32 (*seconds × sampling rate × channels*). The data labels are obtained by prompting participants to complete the self-assessment manikins (Bradley and Lang, 1998) to rate their level of emotional valence, arousal, dominance, and liking after each video clip. A total of 32 × 40 (*participants × video clips*) trails and corresponding labels are included in the dataset.

Establishing evidence has shown that EEG activity is rhythmic (Zheng and Lu, 2015). Such rhythmic patterns are most demonstrable in five frequency sub-bands, which are delta (1–4 Hz), theta (4–8 Hz), alpha (8–12 Hz), beta (12–30 Hz), and gamma (30–45 Hz). Since the DEAP dataset applied a 4–45 Hz band-pass filter after collecting the signals, the delta band is not included in our study.

### 3.2 Data preprocessing

A great body of literature has employed a feature extraction procedure (Subasi, 2007; Liu and Sourina, 2013; Zheng et al., 2017; Fourati et al., 2020b) for EEG signals before classification to improve EEG classification performance. In accordance with previous research (Fourati et al., 2020b), we used the power features of EEG signals for classification, which compute the energy of sub-bands over the energy of the entire frequency band. Our data preprocessing method consists of two procedures:

- Data cropping: first, to generate a sufficiently large dataset, we apply a non-overlapping sliding window for all trails to crop the original EEG samples into smaller data segments. For different classification tasks, we use different lengths of the sliding window. The selection of sliding window size will be explained in detail in section 4.
- Extract frequency bands features: then, the Fast Fourier Transform (FFT) was employed to transform data segments into frequency band features. L2-Normalization is applied to each data segment to reduce the inter-subject variability of EEG signals and prevent overfitting. After data segments were transformed into frequency sub-bands, the energy of each sub-bands is computed using Eq. 1. While a and b are the higher and lower bond of the current frequency band, $F\left(k\right)$ is the normalized frequency band signal. The extracted feature $R\left(\omega \right)$ (Eq. 2) is obtained by dividing sub-band energy by the total band energy. For each channel, we extract four features from four frequency bands. In this manner, each data segment with 32 channels is transformed into a feature vector with 4×32=128 data points.

(1) $$E\left(\omega \right)=\overset{a}{\underset{b}{\int }}F{\left(k\right)}^{2}dk\;\omega \in \Omega ,\Omega =\left\{\theta ,\alpha ,\beta ,\gamma \right\}$$

(2) $$R\left(\omega \right)=\frac{E\left(\omega \right)}{\sum_{f\in \Omega }E\left(f\right)}\;\omega \in \Omega ,\Omega =\left\{\theta ,\alpha ,\beta ,\gamma \right\}$$

### 3.3 Memory capacity

Memory capacity (MC) refers to the network’s ability to store and retrieve past information. It serves as a measure of the echo state network’s capability to store and reconstruct past information. According to the work of Jaeger (2001a,b), memory capacity (Eq. 3) is defined as the maximum possible retrospective of an independently and identically distributed input sequence. Specifically, memory capacity is measured by assessing the correlation between the network output and previous input. For an echo state network with *N* units and identity activation function, the upper bound of its memory capacity is *N*.

The formula for memory capacity is as follows:


(3) $$MC=\overset{\infty }{\underset{k=1}{\sum }}\frac{{\mathrm{cov}}^{2}\left(u\left(t-k\right),y_{k}\left(t\right)\right)}{\mathrm{var}\left(u\left(t\right)\right)\cdot \mathrm{var}\left(y_{k}\left(t\right)\right)}$$

Here, MC represents memory capacity, cov indicates the covariance of two time series, var denotes variance, $u\left(t-k\right)$ is the input shown *k* steps before the current input, and $y_{k}\left(t\right)=w_{k}^{\mathrm{out}}x\left(t\right)=\tilde{u}\left(t-k\right)$ is its reconstruction at the network output, where $w_{k}^{\mathrm{out}}$ is the weight vector of the *k*-th output unit.

### 3.4 M-ESN

#### 3.4.1 ESN backbone

**Figure 2:**
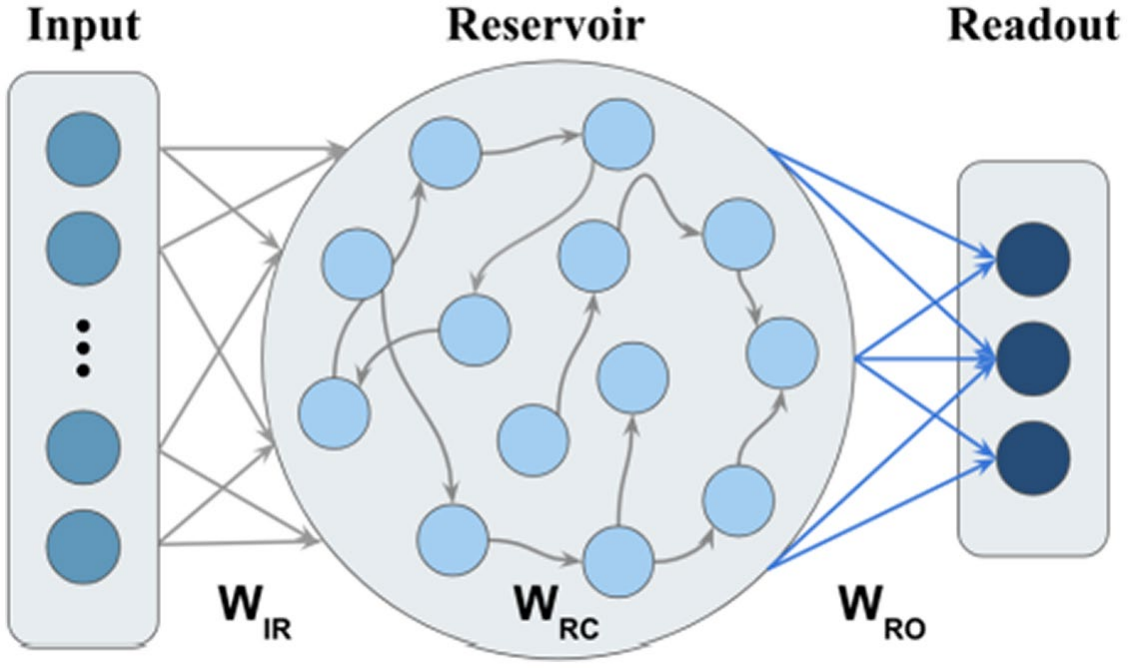
Basic ESN without community structure.

We use ESN with leaky integrator neurons as the model backbone (Jaeger, 2001a,b). In contrast to our proposed M-ESN, it is referred to as basic ESN in the rest of this paper. A basic ESN has three layers: input layer, reservoir, and output layer (Figure 2). The connection weights between the input layer and reservoir, within the reservoir, and between reservoir and readout layer, are denoted by $W_{IR}$ , $W_{RC}$ , and $W_{RO}$ , respectively (Figure 2). $W_{IR}$ , $W_{RC}$ , are randomly generated, while $W_{RC}$ is scaled such that its spectral radius (i.e., the largest absolute eigenvalue of $W_{RC}$ ) $\rho \left(W_{RC}\right)\leq 1$ . This operation is suggested by Lukosevicius and Jaeger (2009) to guard the Echo State Property (ESP) so that information from prior states will be asymptotically washed out. The internal states of the reservoir using Eq. 4 are updated as follows:


(4) $$x\left(t+1\right)=\left(1-\alpha \right)x_{t}+\alpha f_{\mathrm{act}}\left(W_{IR}u_{t}+W_{RC}x_{t}\right)$$

Where $x_{t}$ and $x_{t+1}$ denotes the reservoir states at the time t and t+1 . $f_{\mathrm{act}}\left(\text{.}\right)$ denotes the activation function of the reservoir layer, which usually is a hyperbolic tangent function, and $\alpha \in \left[0,1\right]$ is the leak rate that controls the update speed of the reservoir. $x_{t}$ and its corresponding label $y_{\mathrm{target}}$ at time t are stacked into $X_{rc}$ and $Y_{\mathrm{target}}$ . The readout weights are calculated through ridge regression using Eq. 5, where Ι is an identity matrix and λ is the regularization term introduced to prevent overfitting.


(5) $$W_{RO}=Y_{\mathrm{target}}X_{rc}^{T}{\left(X_{rc}X_{rc}^{T}+\lambda Ι\right)}^{-1}$$

Finally, the predicted classes are obtained using Eq. 6 as follows:


(6) $$y_{\mathrm{pred}}\left(t\right)=W_{RO}x\left(t\right)$$

#### 3.4.2 Modular structure

**Figure 3:**
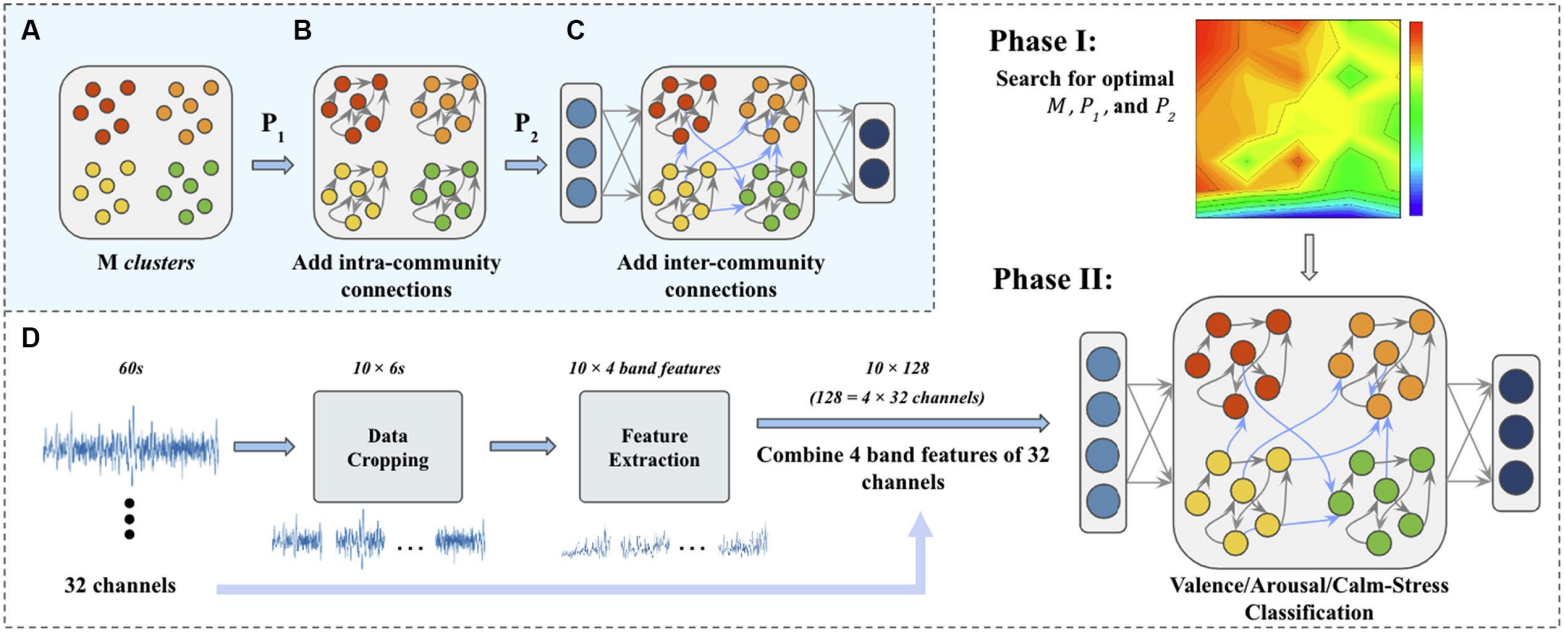
**(A)** N internal neurons are first generated and randomly assigned to M clusters. **(B)** Then, intra-community connections are added according to $P_{1}$ . **(C)** Cross community connections are added according to $P_{2}$ and the reservoir layer is connected to the input layer and the output layer. **(D)** The flow chart of a complete experiment process. In *Phase I*, the optimal parameters are searched in the given parameter space. In *Phase II*, the optimized M-ESN is trained and tested.

Our M-ESN improves basic ESN (Figure 2) by replacing its random reservoir layer with a modular network (Figure 3C). Existing literature introduced a single parameter method (Nematzadeh et al., 2014) to modularize an ESN with two communities (Rodriguez et al., 2021). Under this setting, edges are generated according to a pre-defined global connection density D . Then, μ fractions of edges bridge nodes within the same community and $\left(1-\mu \right)$ fractions of them connect nodes across communities (see Figure 1B). Since the number of modules remains fixed and the inter-community connectivity is dependent on the intra-community connectivity μ , their proposed ESN is optimal in a one-dimensional parameter space. In other words, the ESN is optimized with respect to intra-community connectivity μ .However, as the modular structure is collectively determined by the number of modules, intra-and inter-community connectivity, it may be insufficient to use only one parameter. Thus, we propose in this study to optimize M-ESN with a more comprehensive approach. We use $P_{1}$ to govern the intra-community density and $P_{2}$ to govern global connectivity. $P_{1}$ is the probability of arbitrary two nodes being connected within the same community, while $P_{2}$ governs the likelihood of a link exists between two nodes in different communities. We set $P_{2}$ less than or equal to $P_{1}$ as inter-community connections are often sparser than intra-community connections. When $P_{1}$ equals $P_{2}$ , the reservoir is equivalent to a random network. The modularity M controls the module counts within reservoir. These three parameters together allow more flexible control over modularity and global connectivity, such that our M-ESN is optimized in three-dimensional parameter space ( M , $P_{1}$ , and $P_{2}$ ). A complete generation process of M-ESN is described in Figures 3A–C.

#### 3.4.3 ESN parameters

We use the M-ESN with 600 neurons to perform the experiments. In the previous step, an adjacency matrix based on $P_{1}$ , $P_{2}$ , and M will be generated. Then, connections were added according to that adjacency matrix. We use bidirectional connections such that each connection has a corresponding inverse connection. The weights of these inverse connection pairs are randomly assigned according to a normal distribution within range (0, 1), with approximately 50% of these pairs are in opposite sign. We use a tanh activation function for the reservoir layer. The input weights $W_{IR}$ are uniformly distributed, with values scaled to (−0.1, 0.1), such that the neural dynamics will be more distinguishable by the activation function.

Finally, implementing a modular structure requires the network topology and connection weights to remain unchanged during the course of the training. Consequently, adaptation rules are not included in this study.

## 4 Experiment

We test M-ESN on three tasks, that is, to discriminate emotional valence, arousal, and stress/calm. The DEAP dataset describes emotional status in the following four domains: valence, arousal, dominance, and liking. Each of the four domains is represented by a numerical rating from 1 to 9. In accordance with prior research (Fourati et al., 2020b), we classify numerical values >5 as high arousal/valence (HA/HV) and those ≤5 as low arousal/valence (LA/LV). The calm and stress label is defined collectively by numeric valence and arousal levels, where a signal is classified as stress if valence≤3 and arousal≥5 , and as calm if 4≤valence≤6 and arousal<4 .As discussed in section 3.2, each 60-s trail is sliced into data segments using sliding windows. For the arousal and valence classification task, the sliding window is set to 6 s. However, only 2,790 data segments have a stress/calm labels when using the 6 s sliding window. Thus, a smaller sliding window (*t* = 2 s) is used for stress/calm discrimination task to generate a larger dataset (*n* = 8,370). Our method contains two steps. In the first step, we fix the spectral radius and leakage rate of M-ESN to search for optimal combinations of modules count, local and global connection density on the valence discrimination task. In the second stage, the optimized modular structure is set fixed. We tune the ESN using the leakage rate for different classification tasks. A complete procedure is demonstrated in Figure 3D.

### 4.1 M-ESN optimization

It is well-established that ESN performance is determined by the reservoir topology (Zhang et al., 2011). For M-ESN, the reservoir topology is governed by $P_{1}$ , $P_{2}$ , and M . Hence, in the first phase of the experiment, we fix the spectral radius of M-ESN to 0.85 and the leakage rate α to 0.25, as they work best on basic ESN. Then, we search for the parameters that optimize M-ESN in the three-dimensional discrete parameter space ( M , $P_{1}$ , $P_{2}$ ). The ESN is assessed on the valence discrimination task. The pseudo-code of this process is described in Algorithm 1.

**Figure 4:**
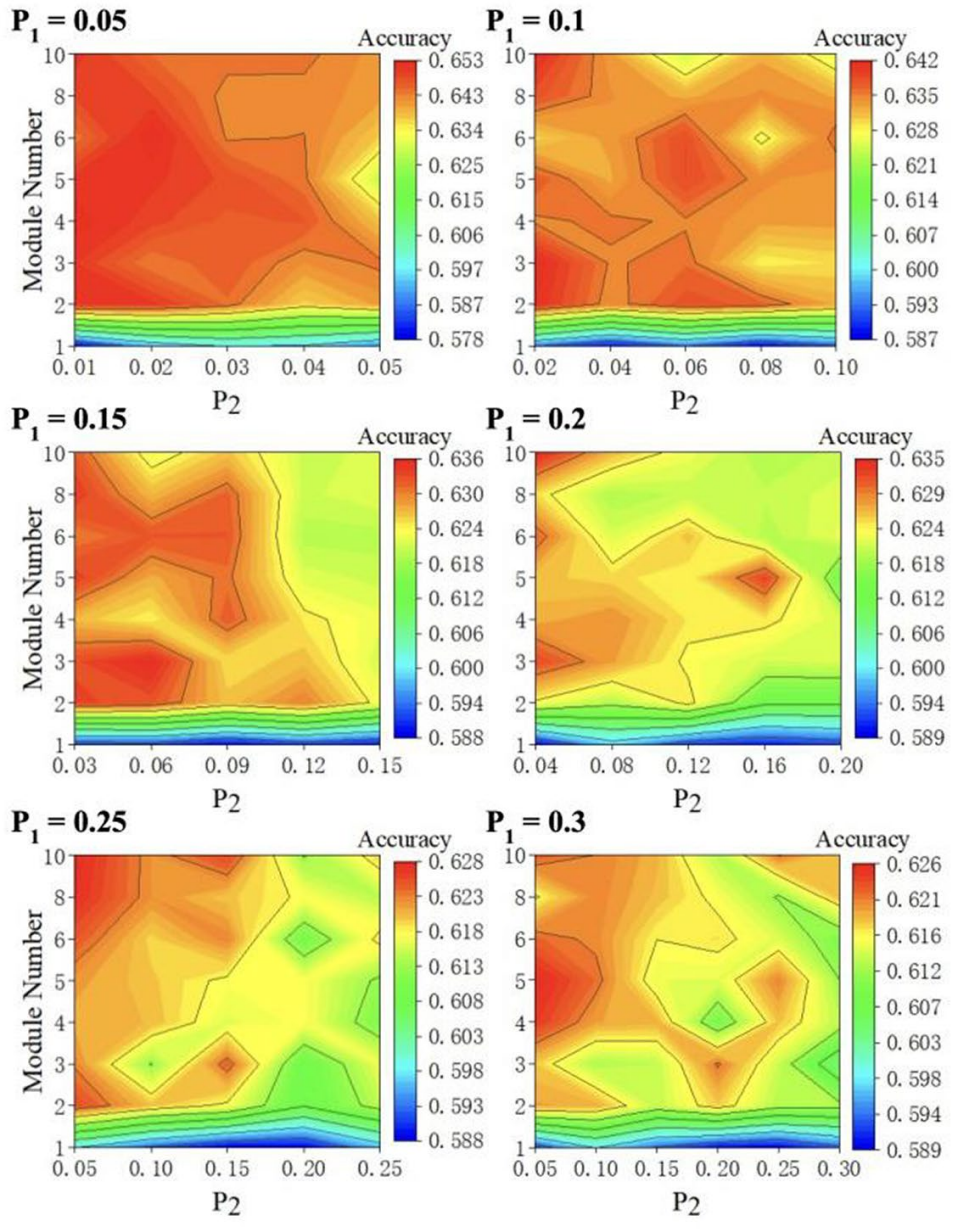
2D accuracy diagram with respect to M and $P_{2}$ at various $P_{1}$ value. The classification accuracies are at its highest value when $P_{1}$ = 0.05, $P_{2}$ = 0.02, and M = 6.

We perform 5-fold cross-validation to reduce the bias introduced by the random train-test split. This 5-fold cross-validation process is conducted by: (1) splitting the dataset into five non-overlapping partitions; (2) leaving one partition out as the test set without repetition and using the remaining four partitions to train the model; and (3) repeating the previous two steps five times and averaging the accuracies. Given that ESN performance is constantly haunted by the random initialization of the reservoir layer, we repeat 5-fold validation three times and average the accuracies. For each $P_{1}$ value, we plot the model performance with respect to M values and $P_{2}$ values in a 2D contour diagram as depicted in Figure 4 to test all parameter combinations iteratively. We plot the 2D diagram using M as *y*-axis and $P_{2}$ as *x*-axis. The highest classification accuracy at each $P_{1}$ value is displayed in Table 1. The model is optimized when $P_{1}$ =0.05, $P_{2}$ =0.02, and M =6.

**Table 1:** Highest valence classification accuracy at each $P_{1}$ value.

$P_{1}$	0.05	0.1	0.15	0.2	0.25	0.3
Acc	0.6526	0.6416	0.6358	0.6352	0.6276	0.6255

The structure and weights of modular reservoir of ESN remain unchanged during training, on the one hand, because this modularized reservoir is optimally designed with optimal classification accuracy, on the other hand, because the reservoir is an RNN structure, its weights are not trained, avoiding the problems of gradient vanishing and gradient explosion, reducing the training complexity of the ESN, and making the ESN simpler to implement, which is also an inherent advantage of ESN.

**Table:** 

Algorithm 1: M-ESN Optimization. **Input**: Modules count M ; Intra-community connectivity $P_{1}$ ; Inter-community connectivity $P_{2}$ ; DEAP dataset. **Output**: Optimal combinations of $P_{1}$ , $P_{2}$ , and M . 1: Let *data* =**normalize** *(data)*. 2: **for** $P_{1}$ = 0.05, 0.1, 0.15, 0.2, 0.25, 0.3 **do** 3: **for** M = 1, 2, 3, 4, 5, 6, 8, 10 **do** 4: **for** $P_{2}=\frac{1}{5}P_{1},\frac{2}{5}P_{1},\ldots P_{1}$ **do** 5: *Acc = MESN(* M , $P_{1}$ , $P_{2}$ ) 6: **if** Acc’ < Acc**then** 7: *Acc’*, M *’*, $P_{1}$ *’*, $P_{2}$ ’ = *Acc*, M , $P_{1}$ , $P_{2.}$ 8: **end for** 9: **end for** 10: **end for** 11: **return** M *’*, $P_{1}$ *’*, $P_{2}$ ’ 12: **end**

### 4.2 Classification results

**Figure 5:**
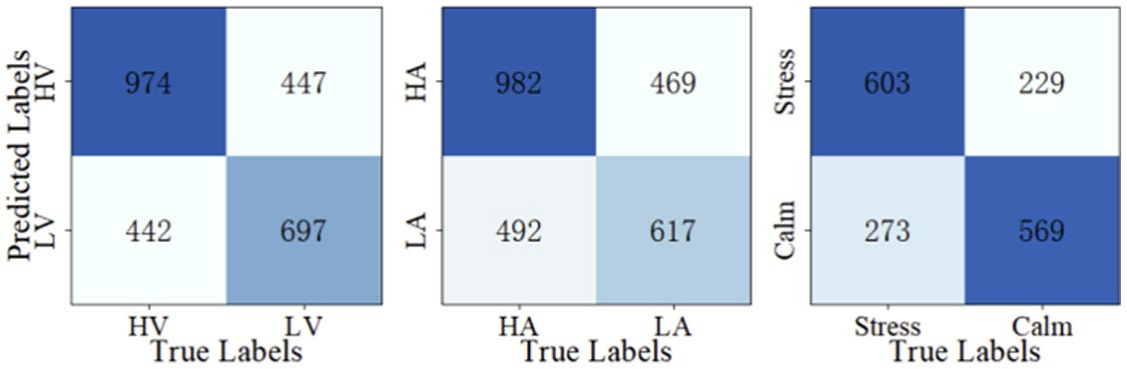
Confusion matrices.

Once the optimal parameters are found, we fix the three modular parameters and fine-tune the M-ESN on different leaking rates for three classification tasks. The optimal leaking rate for valence, arousal, and stress/calm classification are 0.25, 0.3, and 0.25, respectively. Then, we train the M-ESN using the optimal parameter combinations to predict test set labels. We plot the confusion matrix with the predicted classes and their actual labels to calculate the precision, sensitivity, specificity, accuracy, and F1-score (Figure 5). As demonstrated in Table 2, after the modular structure is introduced, we observe an increment in almost all metrics.

**Table 2:** Comparing ESN and M-ESN on three emotion recognition tasks with optimal parameters in terms of precision, sensitivity, specificity, accuracy, and F1-score.

		Precision	Sensitivity	Specificity	Accuracy	F1-score
Valence	ESN	0.639	0.611	0.467	0.594	0.624
	M-ESN	0.686	0.687	0.503	0.653	0.686
Arousal	ESN	0.640	0.581	0.431	0.571	0.608
	M-ESN	0.677	0.666	0.488	0.625	0.671
Stress/Calm	ESN	0.672	0.638	0.464	0.646	0.652
	M-ESN	0.726	0.689	0.458	0.700	0.706

$P_{1}$ = 0.05, $P_{2}$ = 0.02, and *M* = 6.

In Table 3, we compared our results with ESN-IP, ESN-anti-Oja, and ESN-BCM presented by Fourati et al. (2020b). In their work, they provided results trained with raw signals and extracted features. Both kinds of input are trained with three different training schemes. To ensure a fair comparison, we only selected their results trained with frequency band features using the offline mode, which are consistent with our approach. Notably, our results are obtained using M-ESN with the size of 600, while their ESNs have 1,500 internal neurons. In all three tasks, our M-ESN outperforms the plasticity-rules-tuned ESNs with a considerable improvement. This result suggests that the modular structure may provide structural advantages for network performance.

**Table 3:** Comparison of M-ESN classification accuracies with baseline models.

Model	Input signals	ESN size	LA/HA accuracy	LV/HV accuracy	Stress/Calm
ESN-anti-Oja	Features	1,500	0.5977	0.5977	0.6545
ESN-BCM	Features	1,500	0.6172	0.5742	0.6545
ESN-IP	Features	1,500	0.6121	0.5352	0.6906
M-ESN	Features	**600**	**0.6249**	**0.6526**	**0.7001**

Bold notably, our results are obtained using M-ESN with the size of 600, while their ESNs have 1500 internal neurons. In all three tasks, our M-ESN outperforms the plasticity-rules-tuned ESNs with a considerable improvement. This result suggests that the modular structure may provide structural advantages for network performance.

Our results provide several insights. First, as shown in Table 1, the highest prediction accuracy at each $P_{1}$ value decreases monotonically as $P_{1}$ increases. This pattern aligns with previous studies that suggest reservoir sparsity has a significant impact on ESN performance (Zhang et al., 2011). Since the maximum value of $P_{2}$ equals $P_{1}$ , a lower $P_{1}$ will have a lower maximum $P_{2}$ , resulting in a sparser reservoir.

Second, a protruding pattern in our result (Figure 4) is that there appears to be a huge “jump” in its classification accuracy when the number of modules increases from 1 to 2 (Figure 4). As $P_{1}$ , $P_{2}$ , spectral radius, and the leaking rate remain unchanged, we conclude that the introduction of modular structure is responsible for this improvement. Moreover, the best performing M-ESNs have better scores than the best performing basic ESN on almost all evaluation metrics (Table 2).

Furthermore, as depicted in Figure 4, classification accuracy decreases as intercommunity connectivity increases. When $P_{2}=P_{1}$ , the modular structure disappears as inter-community connectivity equals intra-community connectivity. This further support that modular structure contributes to the improved classification accuracy.

### 4.3 Memory capacity

**Figure 6:**
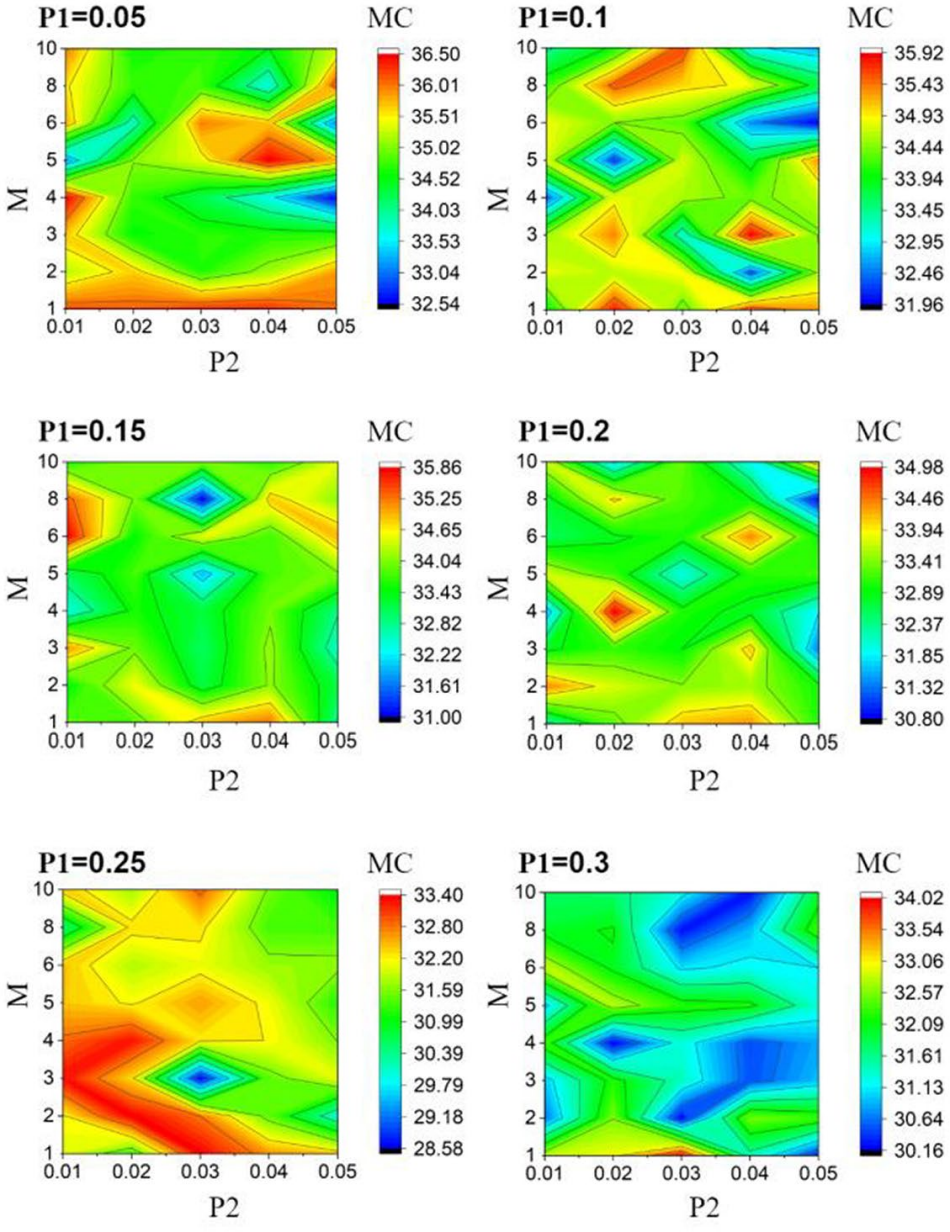
Memory capacity heat map with respect to M and $P_{2}$ at various $P_{1}$ value.

The classification performance of ESNs heavily depends on the encoding capability of the reservoir. In order to provide an explanation for the modular optimization design results shown in Figure 4, we further searched for the optimal values of the module parameters based on the reservoir’s memory capacity as the optimization criterion. As shown in Figure 6, it can be observed that the reservoir achieves the maximum memory capacity when $P_{1}$ = 0.05, $P_{2}$ = 0.04, and M = 5. This optimization result is in close agreement with the results obtained using classification accuracy as the optimization criterion in Figure 4. The discrepancies between the two results can be attributed to certain random factors. Furthermore, when using classification accuracy as the optimization criterion, the weights of the output layer also influence the classification accuracy.

Figure 6 demonstrates that the values of M , $P_{1}$ , and $P_{2}$ have significant impacts on the memory capacity of ESNs. Firstly, overall, a smaller value of the intra-module connection probability $P_{1}$ leads to a larger memory capacity, indicating that a sparser intra-module connectivity promotes the enhancement of memory capacity. Secondly, with an increasing number of modules ( M ), the memory capacity initially increases and then decreases, implying that too many or too few modules are not conducive to memory capacity, and there exists an optimal number of modules. A larger number of modules may result in longer information propagation paths, thereby increasing the signal propagation delay during memory processes and reducing the memory capacity. Additionally, an increasing inter-module connection probability $P_{2}$ also demonstrates an initial increase and then decrease in memory capacity. This phenomenon may be attributed to the fact that when $P_{2}$ is small, its increase benefits information transfer between different modules, while excessively large values of $P_{2}$ weaken the modular characteristics, potentially decreasing the network’s memory capacity. In conclusion, the modularization parameters affect the memory capacity in the same way as the classification accuracy.

### 4.4 Network heterogeneity

**Figure 7:**
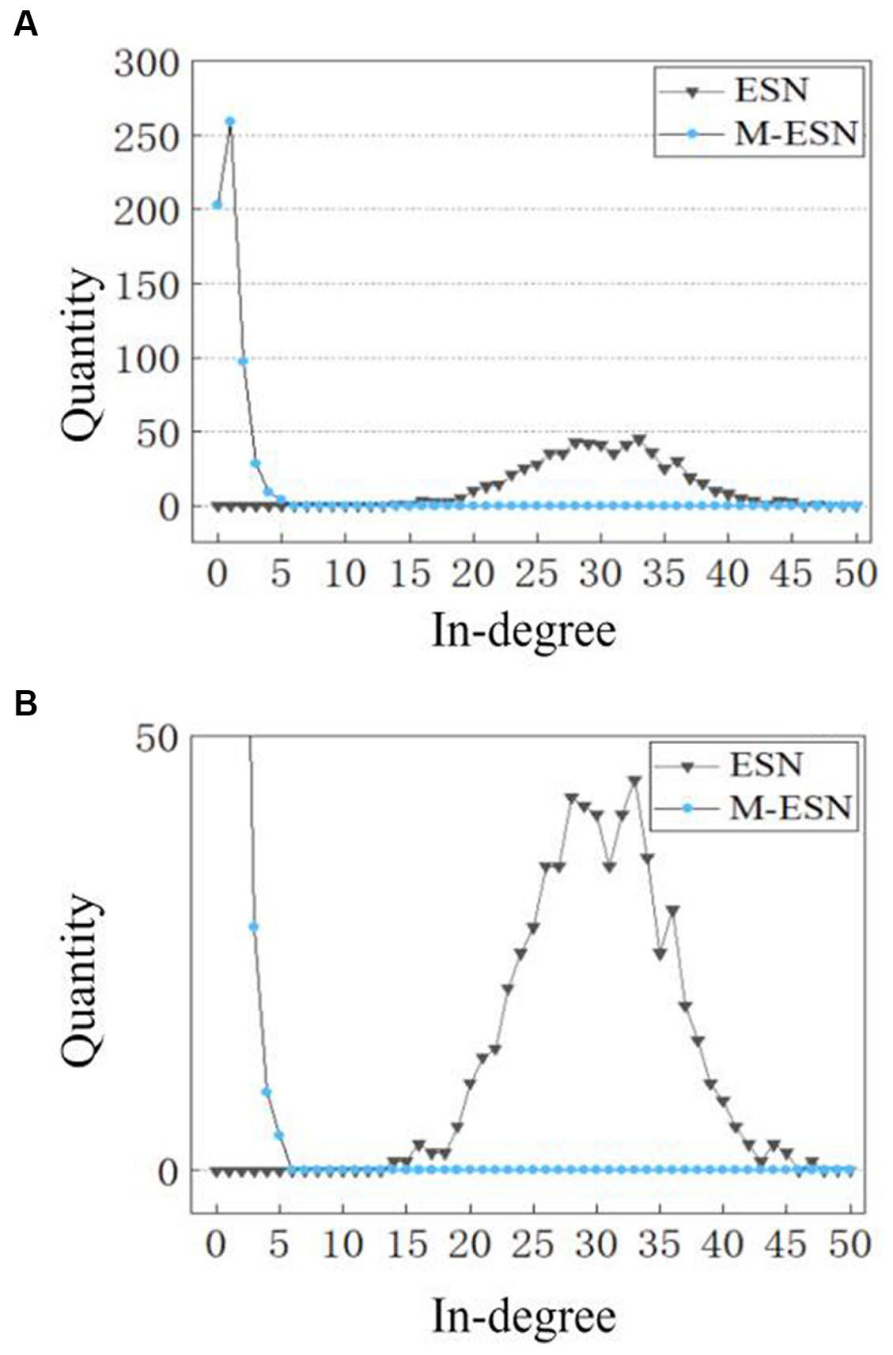
**(A)** In-degree distribution of M-ESN. **(B)** In-degree distribution of M-ESN with zoomed-in *y*-axis. For both ESN and M-ESN: $P_{1}$ = 0.05, *N* = 600. For M-ESN: M = 6.

However, it is still unclear why modular architecture improves network performance. To explain the working mechanism of modular structure in M-ESN, we examine the microscopic changes induced by modular topology, i.e., how modular structure affects neuronal degrees and connection strengths. We found that the modular structure alters the number of in-degree connections for individual neurons. Figure 7 compares the distribution of in-degree connections between a basic ESN and an M-ESN. It appears to be a significant change before and after introducing modular structure. The in-degree connection of most neurons in basic ESN falls between (15, 45), while most neurons in M-ESN have in-degree connection closely distributed around zero.

In Table 4, quantitative results reflect more detailed insights. Although both the variance and the mean of neuron degrees for M-ESN are smaller than basic ESN, the Standard Deviation (SD) over the mean for the M-ESN is significantly larger. Dividing SD by mean is referred to as Coefficient of Variation (CV), which is commonly employed to measure the network heterogeneity in previous literature (Ju et al., 2013; Litwin-Kumar et al., 2017). Comparing to the CV of 5.48 for basic ESN, M-ESN has a CV of 304.17, suggesting M-ESN becomes much more heterogeneous after adopting the community structure.

**Table 4:** Mean, variance, SD, and SD/mean of basic ESN and M-ESN.

	Mean	Variance	SD	SD/Mean
ESN	0.6992	14.6968	3.8336	5.4826
MESN	0.0032	0.9521	0.9758	304.1779

This increased network heterogeneity provides several benefits. First, in the field of neuroscience, it is well-established that neurons in brain are far from being homogeneous (Bullmore and Sporns, 2012; Tripathy et al., 2013). A more heterogeneous network can better simulate biological neural networks. Second, neurons in homogenous networks will yield similar features as they have similar connection weights and degrees. A more heterogeneous network, however, can provide richer features so that they can be more discernable in the hyperspace (Zeldenrust et al., 2021). Similarly, as M-ESN is more heterogeneous, its internal states will be more discernable by ridge regression in the readout layer. This finding is consistent with numerous studies in neuroscience and network science that suggest heterogeneous networks enhance cognitive abilities in brains and model performance in ANNs (Bullmore and Sporns, 2012; Volo and Destexhe., 2021; Zeldenrust et al., 2021).

In addition, a mean in-degree connection close to zero (0.0032) suggests that the majority of neurons within M-ESN have only 1 or 2 incoming connections. A larger CV and variance, however, imply that there are a few neurons that have more than average connection numbers. This network structure with many sparsely connected neurons and a few densely connected cores forms a hub structure. While previous neuroscientific finding (Bertolero et al., 2018) suggests the hub structure facilitates cognitive abilities in human brains, our research extends their studies and proves that hub structure also aids in the efficiency of M-ESN.

Lastly, our finding offers complimentary insights into previous literature (Rodriguez et al., 2021). They indicate increasing modularity will lead to decreasing network performance on ESN using non-step-like activation functions. In our result, however, when modular structure is introduced in our tanh activated M-ESN, we observed a substantial increase in accuracy. We believe this divergence may be task-relevant. Rodriguez et al., 2021 researched a modularized ESN on memory capacity tasks, in which task the model performance is highly dependent on the level of information diffusion within the reservoir. When using step-like activation functions, community structures serve as containers that reduce noise and promote signal. However, since information diffusion will always occur when using non-step-like activation functions, increasing modularity cannot improve the memory capacity in their case. In contrast, we utilized M-ESN for classification tasks, which are highly sensitive to small temporal changes. Non-step-like activation functions broadcast such small changes to the entire reservoir. Consequently, in our experiments, community structures may serve to provide more diverse representations of the original signals, making them more discernable in the hyperspace.

## 5 Discussion and conclusion

A recent study (Kasabov, 2014) proposed a new SNN architecture, called NeuCube, based on a 3D evolving SNN learning from Spatio-and spectro-temporal brain data (STBD) and creates connections between clusters of neurons that manifest chains of neuronal activity. In the present study, the design of modular reservoir structure is also inspired the spatial structure information of the brain network, and we will consider more deeply how to introduce the spatial information into the design of the reservoir in our future research.

FORCE training (Depasquale et al., 2018) is a common method for training ESN, which can train the weights within the reservoir network, as well as the weights of the input and output layers. In this study, drawing on the fact that the connection structure of brain neural networks is modular, the reservoir adopts a modular structure, and its connection structure and weights are determined during initialization, and remain unchanged during training, so modular reservoir retains the advantages of simple training of reservoir computation, and also has a good performance of information processing. FORCE training can be considered in future research to train the weights of the reservoir and used in EEG classification tasks.

In this study, inspired by brain science, we obtained a remarkable improvement in the classification accuracy by adopting the modular structure in ESN for EEG emotion classification. Our work demonstrated the feasibility and superiority of replicating biologically observed structures on RNN to improve model performance. The main reason why the modular ESN performs better than the regular ESN is that its structure is optimized. The reservoir of the regular ESN adopts a randomly connected structure, and the modular ESN adopts the modular structural features of the brain neural network, which improves the performance of the ESN in the EEG classification task. We offered explanations of modularity enhance network performance and reported that neurons become more heterogeneous as the network becomes more modular. The performance enhancement of the modular ESN lies in the stronger structural heterogeneity, and larger coding capacity of the modular reservoir.

The advantage of ESN is that the model size is relatively small and has good performance. The increase of the reservoir size does not necessarily improve the performance of the ESN, too large a reservoir size will saturate the performance of the ESN, and even sometimes lead to overfitting of the ESN. The reservoir of ESN adopts a modularized structure, and the weights inside the reservoir are not trained, which greatly reduces the complexity of ESN training, and the design is relatively simple. Compared to the random reservoir of regular ESN, the modularized reservoir has higher structural heterogeneity and larger coding capacity, thus enhancing improvement of the ESN performance. Future studies could potentially adopt different intra-community connectivity in different communities or seek to reproduce more complex brain structures.

## Data availability statement

The original contributions presented in the study are included in the article/supplementary material, further inquiries can be directed to the corresponding author.

## Author contributions

LY: Writing – original draft, Writing – review & editing. ZW: Writing – original draft, Writing – review & editing. GW: Writing – review & editing. LL: Writing – review & editing. JW: Writing – review & editing. ML: Writing – original draft.
